# Supplementary material for: Midwives’ and Public Health Nurses’ Experiences of Implementing a Guided Version of a Digital Intervention, Mamma Mia, in Maternity and Child Health Care Services: A Reflexive Thematic Analysis
Source: PLOS Digit Health. 2026 Apr 7;5(4):e0001348. doi: 10.1371/journal.pdig.0001348 (PMC13056171; doi:10.1371/journal.pdig.0001348)
Supplement: S1 File — (DOCX) [file pdig.0001348.s001.docx]

| Theme | Codes | Transcribed text: Norwegian | Translation | Memo |
| --- | --- | --- | --- | --- |
| *“****Broken Expectations – Navigating the Training-Practice Gap”*** | Does not feel ownership of the work with Mamma Mia  Was positive about participating in the beginning, but motivation dropped  Doesn't feel like I've really gotten started with Mamma Mia  Hard to know what the best way is to motivate to use Mamma Mia  Mamma Mia more extensive than expected | «Jeg har ikke funnet ut hva som er den lureste måten å [motivere til videre bruk] på nei.»  «Jeg satt igjen og tenkte sånn: gjør jeg egentlig ingenting av det jeg skal? For jeg vet ikke hva jeg gjør. Og så skal jeg velge mellom hvilke fire teknikker jeg bruker når jeg veileder, også føler jeg egentlig at jeg bare snakker. Jeg vet ikke hva det kalles, ikke sant?»  «… at jeg skal være en slags innsalg for et produkt som jeg ikke har vært med selv på å kunne forme eller… eller å eie på en måte. Jeg føler ikke noe eierskap, og jeg har vært... Jeg kunne ønske at vi kunne ha kommet mer og mer sånn innspill til hvordan fungerer dette?»  «Og hvis jeg skal oppsummere, så var jeg veldig positiv til prosjektet sånn i utgangspunktet, for jeg synes det var så utrolig viktig tema med psykisk helse i svangerskapet. Men jeg er altså blitt litt sånn sitter med en litt sånn for stor, litt for overveldende oppgave.»  «Jeg følte liksom at gud det her tar mye tid, og jeg må konsentrere meg, dette vil kreve mye da. Men mens det i praksis egentlig ikke er det, så har det kanskje virket mer komplisert enn det det egentlig er.» | "I haven't figured out the smartest way to [motivate further use], no."  "I was left thinking: am I really not doing anything of what I'm supposed to do? Because I don't know what I'm doing. And then I have to choose between which four techniques I use when guiding, and I really feel like I'm just talking. I don't know what it's called, right?"  “… that I should be a kind of salesperson for a product that I have not been involved in being able to shape or... or to own in a way. I don't feel any ownership, and I've been... I wish we could have come up with more and more input like this, how does this work?"  “I was very positive about the project in the first place, because I think it was such an incredibly important topic… mental health in pregnancy. But now I’m like… sitting with a task that is a little too big, a little too overwhelming."  "I kind of felt that god this takes a lot of time, and I have to concentrate, this will take a lot then. But while in practice it really isn't, it may have seemed more complicated than it really is." | The intervention was perceived as complex, with unclear procedures.  Expectations for a more systematic approach (beyond just recommending the app) were unmet.  Lack of hands-on, skills-based training created frustration and demotivation.  Benefits of training and supervision varied; some had not completed self-study beforehand.  Most public health nurses lacked prior experience, while only a few midwives had extensive experience.  Expectations for own role in guidance were unclear.  Participants reported broken expectations, frustration, and demotivation linked to training, unclear roles, and added workload. |
| ***Balancing Belief and Doubt – Navigating digital care in relational professions*** | Feels negative to encourage mobile use  Skeptical about whether an app should replace traditional guidance/treatment/health personnel  Ambivalence to integrate digital tools  Content vs Design  Positive to research-based interventions  Many women find that they do not want to participate in the study or use Mamma Mia  Difficult to recruit due to a lot of text and information  Difficult to recruit due to technical problems  Hard to feel that you are pressuring pregnant women to use the app  Challenging to recruit due to late consultations during pregnancy and low birth rates  Bad feedback creates less motivation for implementation  Generally positive about the app Mamma Mia  Mamma Mia promotes health by increasing awareness of one's own resources  Mamma Mia as a concrete tool  Positive experience with Mamma Mia prior to participation in the study  The Mamma Mia app is convenient  Positive to Mamma Mia as a supplement to the health centre service  Mamma Mia can be a gateway to talking about mental health and other topics  Mamma Mia more accessible mental health interventions | «Er jo så viktig at det vi jobber med er forskningsbasert.»  «En mer moderne måte… fordi vi engasjerer kvinnene på en annen måte, enn en sånn enveis kommunikasjon som vi ofte har da. Vi informerer og skal jo gjennom en smørbrødliste med informasjon, så tenker jeg, kanskje det ikke er sånn man skal jobbe. Kanskje vi heller skal undre seg litt mer sammen».  «Så jeg tror absolutt at man er inne på noe når man tenker at psykisk helse må implementeres i digitale og appenes verden. Så er det bare dette med vår rolle opp i det.»  «jeg er ikke så veldigglad i å oppfordre til enda mer bruk av telefonen i et hverdagsliv da.»  «For meg er det jo litt sånn … Men det er sikkert fordi jeg er godt voksen [...] altså det er enda en ting man skal sitte på telefonen med da. Telefonen blir liksom så stor del […] det er nesten en forlengelse av armen til mange mennesker, og det skal liksom … Dette skal også inn på … På telefon […] Jeg vet jo ikke helt … Hvordan de opplever det alltid. Hvordan, hvor hensiktsmessig den er eller hvor mye til hjelp den er da. Kanskje kontra støttesamtaler…»  «Hva skal jeg si, litt sånn blandet, fordi det er så viktig tema, og hvis det kan være et fint verktøy for de så er det kjempebra. Men jeg synes på en måte, det skal jo ikke […] erstatte oss. Jeg er […] generelt litt skeptisk til apper. Men [jeg skal] være åpen hvis den kan være et hjelpemiddel for de gravide.»  «Men jeg har skjønt at det har kommet for å bli men et alternativ da et supplement til vår tilstedeværelse.»  «…For meg ville det aldri noen gang kunne erstatte møtet mellom mennesker.»  «De bruker mye digitale hjelpemidler, så sånn sett så er det helt midt i blinken egentlig.»  «Men [Mamma Mia] skaper litt sånn der frustrasjonen rundt det som vi allerede har. Fordi at vi ikke har nok oppfølging å tilby … Til de som sliter da. […] vi har ikke psykologer i kommunen.»  «Jeg synes egentlig det viktigste for min del er jeg synes at Mamma Mia appen er bra verktøy»  «Mamma Mia er et mer konkret, mer tilgjengelig og praktisk verktøy, som er mer persontilpasset og kan bidra til god bevisstgjøring av gravides egne ressurser». | "It's so important that what we work with is research-based"  "A more modern way... Because we engage the women in a different way, than the one-way communication that we often have then. We inform and have to go through a list of information, then I think, maybe that's not how you should work. Maybe we should rather reflect more together."  "So, I definitely think that you are on to something when you think that mental health needs to be implemented in the world of apps. Then there's just this thing about our role in it."  "'m not very fond of encouraging even more use of the phone in everyday life."  “For me, it's a bit like that ... But it's probably because I'm well grown up [...] so that's another thing you must sit on the phone with then. The phone becomes such a big part [...] it's almost an extension of many people's arm, and it's supposed to be ... This will also be on ... On the phone [...] I don't really know... How they experience it. How, how appropriate it is or how much of a help it is then. Maybe versus counselling..."  "What can I say, a bit mixed, because it's such an important topic, and if it can be a nice tool for them, that's great. But I think in a way, it should not [...] replace us. I'm [...] generally a little skeptical about apps. But [I will] be open if it can be an aid for the pregnant women."  "But I have realized that it has come to stay, but an alternative then, a supplement to our presence."  «… For me, it would never, ever replace the meeting between people."  "But [Mamma Mia] creates a bit of frustration around what we already have. Because we don't have enough follow-up to offer... To those who are struggling then. […] We do not have psychologists in the municipality."  "I think the most important thing for me is I think that the Mamma Mia app is a good tool"  "Mamma Mia is a more concrete, more accessible and practical tool, which is more personalised and can contribute to good awareness of pregnant women's own resources." | National guidelines encourage addressing parental mobile use and parent–child interaction.  Ongoing public and professional debate about parents’ screen use creates dilemmas: recommend, discourage, or promote limited screen time.  Cultural differences in openness about mental health influenced engagement.  Recruitment strategies involved selective offering (based on perceived receptiveness and capacity) to reach study targets quickly.  Time demands and workload were highlighted.  Importance of the relationship between healthcare professionals and pregnant women.  Dilemma between motivating/recommending vs. pressuring women to participate.  Professionals noted differing knowledge levels and attitudes among both staff and women.  Curiosity about moving from one-way information-giving toward open dialogue.  Alignment with health service values: autonomy, family focus, health promotion, and prevention.  Potential synergy between Mamma Mia and existing services.  Recognition that most health-promoting and preventive work occurs in women’s everyday lives, not solely through health services.  Mixed views: both skepticism and openness.  Skepticism often related less to Mamma Mia itself and more to digital tools in general and their implications for professional practice. |
| **“Learning by Doing and Support: Future Optimism”** | Office professional keeps track of participants in Mamma Mia  Transfer of Mamma Mia participants in collaboration meetings  "Selling" Mamma Mia in consultations by linking it to mental health  Check on home visits who is a participant in Mamma Mia to remind them of use  Management facilitates and supports the work with Mamma Mia  Close collaboration  Not very resource-intensive  Continuity and coherent services  Mamma Mia appears to be more complicated than it "really is"  Expectations for a new practice | «Ja vår leder har […] vært veldig positivt og […] har veldig troen på Mamma Mia, og er med i styringsgruppe ho også…»  «Noen ganger så har jeg bare ventet […] [og] tatt [det] opp på et senere tidspunkt. Fordi at sånn som […] sa litt, det første hjemmebesøket vi har, så er det jo ofte litt brannslukning, fordi de har kommet hjem og livet [plutselig er] snudd helt på hodet, så denne appen er kanskje det siste de tenker på, selv om det har nytte selvfølgelig, men kanskje ikke akkurat der og da, asså de to tilfellene jeg har vært borti nå da, det er bare noen, så da har jeg ventet...»  «det kan lede inn til veldig gode samtaler merker jeg, når vi har tid til det.»  «For helsesykepleiere og jordmødre at vi har litt flere sånne treffpunkter å gjøre noe felles… hvordan vi skal sikre gode overganger mellom svangerskap og barsel. Spesielt de mest sårbare mødrene. I teorien da, så får vi se om det går sånn i praksis.... Jeg tenker i hvert fall er en fordel for helsestasjonen og mye av det vi holder på med allerede».  «Men det jeg trengte å trekke fram også som en fordel, er jo at det er noe som er kontinuerlig fra svangerskapet over i barsel. Det synes jeg jo har vært fint å få noe som er liksom er konkret og som er litt sånn konsistent da gjennom svangerskap og barsel så er det blitt en litt sånn samarbeidsarena».  «For det var litt sånn overveldende i starten synes jeg det å skulle finne fram til disse tingene, men det det er ganske oversiktlig, tenker jeg. Det går veldig greit.» | "Yes, our leader has [...] has been very positive and [...] has a lot of faith in Mamma Mia, and is part of the steering group she too..."  "Sometimes I've just waited [...] [and] brought [it] up at a later date. Because as [...] said a little, the first home visit we have, it's often a bit of firefighting, because they've come home and their lives are [suddenly] turned completely upside down, so this app might be the last thing on their minds, even though it's useful of course, but maybe not right then and there, like the two cases I've come across now, There are only a few, so then I have been waiting..."  “it can lead to very good conversations, I notice, when we have time for it.”  'For public health nurses and midwives, we have a few more such meeting points to do something in common... how to ensure good transitions between pregnancy and maternity. Especially, the most vulnerable mothers. In theory then, then we will see if it goes like that in practice... I think is at least an advantage for the MCHC and a lot of what we are already doing."  'But what I needed to highlight as an advantage is that it is something that is continuous from pregnancy to postnatal care. I think it has been nice to get something that is kind of concrete, and is a bit consistent, through pregnancy and maternity it has become a bit of a collaboration arena."  "Because it was a bit overwhelming in the beginning, I think it had to find these things, but it's pretty clear, I think. It's going very smoothly." | Need for clear strategies to ensure continuity and overview in daily work.  Use of Mamma Mia integrated into both consultations and home visits.  Experience shows that practical implementation is less demanding than training initially suggested.  Importance of leadership support, collaboration, and resource management.  Perception of shared decision-making differed between small health centers and larger municipalities.  Supervision helped maintain motivation, clarify roles, and support staff in adapting practices.  Variation in how support materials were used; reliance decreased with more experience.  Guidance sessions also provided professional input and deeper knowledge of Mamma Mia.  Motivational aspects of guidance were emphasized.  Introduction and guidance were tailored to the needs and circumstances of individual women when gaining experience. |
