# Supplementary material for: Midwives’ and Public Health Nurses’ Experiences of Implementing a Guided Version of a Digital Intervention, Mamma Mia, in Maternity and Child Health Care Services: A Reflexive Thematic Analysis
Source: PLOS Digit Health. 2026 Apr 7;5(4):e0001348. doi: 10.1371/journal.pdig.0001348 (PMC13056171; doi:10.1371/journal.pdig.0001348)
Supplement: Attachment — Submitted filename: Response to Editor.docx [file pdig.0001348.s003.docx]

Dear Kate Anne Manalo Dimayuga
PLOS Digital Health,

PDIG-D-25-00667
Midwives’ and Public Health Nurses’ Experiences of Implementing a Guided Version of a Digital Intervention, Mamma Mia, in Maternity and Child Health Care Services: A Reflexive Thematic Analysis

We've addressed the following issues:

1. Please ensure that the Author List in your manuscript file matches the Author List in the online submission form, including initials and order. Currently, author "Thea Grossmann SUNDREHAGEN" name listed in manuscript file is different from online submission form.
Please use either initials or full names in both the manuscript and online submission form.

**AR:** The manuscript has been updated and match the Author List.

2. Kindly confirm if you are submitting a clinical trial, it must be registered in a recognized international registry, e.g. ClinicalTrials.gov or the International Standard Randomized Controlled Trial Number (ISRCTN) Registry.

Please include the name of the registry, the trial number, and the trial URL in the Methods section of your manuscript file.

**AR:** We’ve updated the Methods section to clarify.

Please also include all of the following with your submission:

1) Clinical Trials Registration Number (usually NCT or ISRCTN number) present in the Abstract section of the Manuscript file;

**AR:** The ISRCTN number is corrected as it had a typo. ISRCTN nr. 39971. And the Abstract section has been updated.

2) A CONSORT 2025 Checklist or relevant reporting guideline (uploaded as a Supporting Information file)

**AR:** This is not applicable to this study.

3) CONSORT Flow Diagram (uploaded as Fig 1);

**AR:** This is not applicable to this study.

4) A Trial Protocol - please provide the most recently approved version, including any amendments, with the date of the change noted (uploaded as a Supporting Information file);

**AR:** The most recently approved version has been uploaded as Supporting Information.

5) Details of prior approval for human subjects research by an institutional review board (IRB) or equivalent ethics committee(s) in the Methods section, including board or committee names and approval numbers.

**AR:** The most recently approved version has been uploaded as Supporting Information, and the Methods section has been updated with this information.

We also recommend that authors of clinical trial studies provide a SPIRIT 2025 checklist. You can read more about the 2025 SPIRIT updates and access a copy of the checklist at: [https://eur01.safelinks.protection.outlook.com/?url=https%3A%2F%2Fwww.consort-spirit.org%2F&data=05%7C02%7Ceso%40r-bup.no%7C6a32058d35584c0f92aa08dde7aa88cc%7Ca34f6ee52cae4a46a7796480cace54ec%7C0%7C0%7C638921441833507974%7CUnknown%7CTWFpbGZsb3d8eyJFbXB0eU1hcGkiOnRydWUsIlYiOiIwLjAuMDAwMCIsIlAiOiJXaW4zMiIsIkFOIjoiTWFpbCIsIldUIjoyfQ%3D%3D%7C0%7C%7C%7C&sdata=EiI9DDpZGCgoTsmxRNQ4dfGGizJw6szBW0gJGeNCzoU%3D&reserved=0](https://urldefense.com/v3/__https://www.consort-spirit.org/__;!!M-1nqGDpNA!53gIEkI7kc3XYNQCHwDcNuri8s36IiKuNE__PalxP4j0V-4ac89NiuINv8WBGm0mJnQAy6LHDRgtkA1O9oxwEic$).

**AR:** This is not applicable to this study.

Thank you for your feedback.

Kind regards,

Ellen Solstad Olavesen
