# Supplementary material for: Midwives’ and Public Health Nurses’ Experiences of Implementing a Guided Version of a Digital Intervention, Mamma Mia, in Maternity and Child Health Care Services: A Reflexive Thematic Analysis
Source: PLOS Digit Health. 2026 Apr 7;5(4):e0001348. doi: 10.1371/journal.pdig.0001348 (PMC13056171; doi:10.1371/journal.pdig.0001348)
Supplement: Attachment — Submitted filename: Manuscript Review.docx [file pdig.0001348.s004.docx]

**REVIEW OF MIDWIVES’ AND PUBLIC HEALTH NURSES’ EXPERIENCES OF IMPLEMENTING A GUIDED VERSION OF A DIGITAL INTERVENTION, MAMMA MIA, IN MATERNITY AND CHILD HEALTH CARE SERVICES: A REFLEXIVE THEMATIC ANALYSIS**

**Abstract**

The abstract addresses an important gap in understanding how digital mental health tools are integrated into maternity care, but it reads more like a summary than a true reflection of thematic insight. For a reflexive thematic analysis, the themes should do more than describe. They should interpret professional meaning, tension, or change, showing how midwives and nurses made sense of the digital shift. The three themes are great but the authors should do more than listing without enough interpretive context to show the analytical depth behind them. The conclusion should also highlight how these findings advance implementation theory or digital health practice in relational care settings.

**Introduction**

The introduction covers the right ground but takes too long to get to the point. It does a good job explaining why perinatal mental health and digital tools like Mamma Mia matter, but it buries the actual research gap under too much background and policy detail. The global and Norwegian context is useful, but it should quickly lead to why midwives’ and nurses’ experiences with guided digital care are worth studying. The focus needs to shift from what Mamma Mia is to what’s not yet understood about putting it into real practice.

The section on training, the EMS model, and the structure of the program feels out of place here—it belongs in methods. The introduction should stick to setting up the problem: that digital interventions in relational care face cultural, practical, and professional barriers, and that this study helps unpack that.

Great introduction from the authors. It’s well-written and evidence-based but needs improvement about “what the system does,” more “why this study matters right now.”

**Materials and Methods**

The methods part is great and shows strong attention to rigor from the authors. The use of reflexive thematic analysis fits the study’s aim well, and the team clearly put effort into ensuring transparency and reflexivity. The COREQ checklist, ethical approvals, and investigator triangulation all strengthen credibility. Still, some parts sound too procedural, more like instructions than explanation. The section could focus more on why certain choices were made and how they shaped the analysis, not just what was done. The use of CFIR is smart, but it would help to explain how it guided, rather than limited, the inductive process. The rigor section stands out as a strength, though it could connect better to how reflexivity actually influenced interpretation, not just list steps taken.

The authors can improve this section with this.

**Results**

The results section presents a well-organized and coherent thematic structure that captures the real-world complexity of implementing a guided digital intervention in relational care settings. The first theme, *“Broken Expectations – Navigating the Training-Practice Gap,”* is particularly strong because it surfaces a core implementation barrier that often gets overlooked — the mismatch between abstract training and applied clinical reality. The participants’ frustration feels authentic and highlights how inadequate preparation can undermine confidence and motivation. However, the analysis could go deeper into how this “gap” shaped participants’ later attitudes toward the intervention. The authors rightly connect this to timing and context, but they could strengthen the argument by tying it to established implementation science concepts like readiness or fidelity. The inclusion of quotes adds richness, though the section could more clearly separate emotional reactions from systemic barriers.

The second theme*, “Balancing Belief and Doubt – Navigating Digital Care in Relational Professions,”* does an excellent job illustrating professional ambivalence. The tension between relational care and digital delivery comes through clearly and adds depth to the findings. Still, the theme occasionally drifts toward description rather than interpretation, it reports what participants felt without the “why” these beliefs persist in a system increasingly adopting digital tools. The theme would be better if it connected these doubts to broader frameworks like professional identity theory or digital trust in healthcare. The insight about digital interventions potentially conflicting with relational values is powerful, and situating it more critically within existing literature would enhance its theoretical contribution.

The final theme, *“Learning by Doing and Support – Future Optimism,”* brings a much-needed sense of progression and hope. The transition from skepticism to cautious acceptance is realistic. The emphasis on practical experience, peer collaboration, and ongoing supervision as drivers of confidence is compelling and aligns with real-world implementation dynamics. However, the discussion of optimism could be balanced with a reflection on sustainability: will this confidence last once research support ends? Still, the theme effectively captures the adaptive learning process and the evolving professional buy-in, making it the most forward-looking and policy-relevant part of the analysis.

**Discussion**

The discussion is well thought out and connects the findings clearly, but it leans too much on theory and could feel more grounded in real experiences. The part about the training–practice gap is strong, but it would help to show what could actually make future training more useful or realistic for midwives and nurses. The section on balancing digital tools with relational care hits an important point, though it would come across better if it focused more on what participants said and felt instead of citing frameworks. The ending on learning by doing works well, it shows growth, confidence, and teamwork, but the whole discussion would feel more genuine if it spoke more from the participants’ perspective and less from a theoretical distance.

Thank you.
