# Supplementary material for: Midwives’ and Public Health Nurses’ Experiences of Implementing a Guided Version of a Digital Intervention, Mamma Mia, in Maternity and Child Health Care Services: A Reflexive Thematic Analysis
Source: PLOS Digit Health. 2026 Apr 7;5(4):e0001348. doi: 10.1371/journal.pdig.0001348 (PMC13056171; doi:10.1371/journal.pdig.0001348)
Supplement: Attachment — Submitted filename: Point-by-point response to reviewers and editor PLOS ONE.docx [file pdig.0001348.s005.docx]

# **EDITORIAL REPORT AND REVIEWER COMMENTS**

Thank you for submitting your manuscript to PLOS Digital Health. After careful consideration, we feel that it has merit but does not fully meet PLOS Digital Health's publication criteria as it currently stands. Therefore, we invite you to submit a revised version of the manuscript that addresses the points raised during the review process. We look forward to receiving your revised manuscript.

**AUTHOR RESPONSE TO EDITOR AND REVIEWERS**

We thank the Editor and reviewers for their careful evaluation of our manuscript and for the constructive and insightful feedback provided. We have revised the manuscript thoroughly in response to all comments. Changes are shown in the revised manuscript with tracked changes. Below, we address each comment point by point.

**Reviewer Responses to Questions**

**2. Has the statistical analysis been performed appropriately and rigorously?**

**Reviewer #1:** Yes
**Reviewer #2:** N/A
**Reviewer #3:** No

**Author Response:**
Thank you for this comment. We have revised the Methods section to clarify how we handled demographic variables. The revised text now specifies the variables included and states that they are presented as descriptive statistics (frequencies, percentages, and means), and that they are analyzed using SPSS version 27.0.

**Review Comments to the Author**

**Reviewer #1:** This is a well-conducted and timely qualitative study exploring midwives’ and public health nurses’ experiences with implementing a guided digital intervention in perinatal care. The reflexive thematic analysis is rigorous, the methods transparent, and the findings provide valuable insight into the real-world challenges of translating digital health tools into relational care settings. The discussion effectively connects the themes to implementation science frameworks, though it would benefit from a sharper balance between theory and participants’ lived experiences, and a stronger focus on actionable strategies for training, supervision, and leadership support. See my attached detailed review.

**Author Response:**
Thank you for this positive and constructive assessment. We have revised the manuscript to strengthen the balance between theoretical framing and participants’ lived experiences, and to sharpen the focus on actionable implications for training, supervision, and leadership support. Specific revisions are detailed below in response to your individual comments.

**Reviewer #1:**

**Abstract**

The abstract addresses an important gap in understanding how digital mental health tools are integrated into maternity care, but it reads more like a summary than a true reflection of thematic insight. For a reflexive thematic analysis, the themes should do more than describe. They should interpret professional meaning, tension, or change, showing how midwives and nurses made sense of the digital shift. The three themes are great but the authors should do more than listing without enough interpretive context to show the analytical depth behind them. The conclusion should also highlight how these findings advance implementation theory or digital health practice in relational care settings.

**Author Response:**
We have revised the abstract to strengthen its interpretive emphasis. The revised version clarifies how the themes capture professional meaning-making, tension, and change, and more explicitly articulates the study’s contribution to implementation theory and digital health practice in relational care settings.

**Introduction**

The introduction covers the right ground but takes too long to get to the point. It does a good job explaining why perinatal mental health and digital tools like Mamma Mia matter, but it buries the actual research gap under too much background and policy detail. The global and Norwegian context is useful, but it should quickly lead to why midwives’ and nurses’ experiences with guided digital care are worth studying. The focus needs to shift from what Mamma Mia is to what’s not yet understood about putting it into real practice.

The section on training, the EMS model, and the structure of the program feels out of place here—it belongs in methods. The introduction should stick to setting up the problem: that digital interventions in relational care face cultural, practical, and professional barriers, and that this study helps unpack that.

Great introduction from the authors. It’s well-written and evidence-based but needs improvement about “what the system does,” more “why this study matters right now.”

**Author Response:**
We have revised the introduction in line with this feedback. The narrative has been tightened, descriptive policy detail reduced, and the focus shifted toward what remains insufficiently understood about implementing guided digital interventions in relational care. Descriptions of the intervention structure, training, and EMS model have been moved to the Methods section.

**Materials and Methods**

The methods part is great and shows strong attention to rigor from the authors. The use of reflexive thematic analysis fits the study’s aim well, and the team clearly put effort into ensuring transparency and reflexivity. The COREQ checklist, ethical approvals, and investigator triangulation all strengthen credibility. Still, some parts sound too procedural, more like instructions than explanation. The section could focus more on why certain choices were made and how they shaped the analysis, not just what was done. The use of CFIR is smart, but it would help to explain how it guided, rather than limited, the inductive process. The rigor section stands out as a strength, though it could connect better to how reflexivity actually influenced interpretation, not just list steps taken.

The authors can improve this section with this.

**Author Response:**
Thank you for this constructive feedback. We have revised the Methods section to better explain the rationale behind key methodological choices, including how CFIR informed the inductive analytic process. We also strengthened the rigor section by more explicitly linking reflexivity to interpretation rather than listing procedural steps.

**Results**

The results section presents a well-organized and coherent thematic structure that captures the real-world complexity of implementing a guided digital intervention in relational care settings. The first theme, *“Broken Expectations – Navigating the Training-Practice Gap,”* is particularly strong because it surfaces a core implementation barrier that often gets overlooked — the mismatch between abstract training and applied clinical reality. The participants’ frustration feels authentic and highlights how inadequate preparation can undermine confidence and motivation. However, the analysis could go deeper into how this “gap” shaped participants’ later attitudes toward the intervention. The authors rightly connect this to timing and context, but they could strengthen the argument by tying it to established implementation science concepts like readiness or fidelity. The inclusion of quotes adds richness, though the section could more clearly separate emotional reactions from systemic barriers.

**Author Response:**
We have revised this theme to more explicitly examine how the training–practice gap influenced participants’ subsequent confidence, motivation, and engagement with the intervention. These findings are now more clearly linked to implementation concepts such as readiness for change and feasibility in the Discussion. We have also clarified the distinction between emotional reactions and systemic barriers.

The second theme*, “Balancing Belief and Doubt – Navigating Digital Care in Relational Professions,”* does an excellent job illustrating professional ambivalence. The tension between relational care and digital delivery comes through clearly and adds depth to the findings. Still, the theme occasionally drifts toward description rather than interpretation, it reports what participants felt without the “why” these beliefs persist in a system increasingly adopting digital tools. The theme would be better if it connected these doubts to broader frameworks like professional identity theory or digital trust in healthcare. The insight about digital interventions potentially conflicting with relational values is powerful, and situating it more critically within existing literature would enhance its theoretical contribution.

**Author Response:**
We have strengthened the interpretive focus of this theme by foregrounding how participants’ ambivalence was shaped by professional values related to relational care, clinical judgement, and role responsibility. While the Results retain an empirical emphasis, broader theoretical interpretation—drawing on professional identity and trust in digital care—is developed more fully in the Discussion.

The final theme, *“Learning by Doing and Support – Future Optimism,”* brings a much-needed sense of progression and hope. The transition from skepticism to cautious acceptance is realistic. The emphasis on practical experience, peer collaboration, and ongoing supervision as drivers of confidence is compelling and aligns with real-world implementation dynamics. However, the discussion of optimism could be balanced with a reflection on sustainability: will this confidence last once research support ends? Still, the theme effectively captures the adaptive learning process and the evolving professional buy-in, making it the most forward-looking and policy-relevant part of the analysis.

**Author Response:**
We agree that sustainability is a key consideration. We have revised this theme to frame optimism as conditional and support-dependent, rather than stable or guaranteed. The Results now emphasize that growing confidence was closely tied to supervision, peer collaboration, and organizational scaffolding. Broader sustainability implications beyond the research period are addressed in the Discussion.

**Discussion**

The discussion is well thought out and connects the findings clearly, but it leans too much on theory and could feel more grounded in real experiences. The part about the training–practice gap is strong, but it would help to show what could actually make future training more useful or realistic for midwives and nurses. The section on balancing digital tools with relational care hits an important point, though it would come across better if it focused more on what participants said and felt instead of citing frameworks. The ending on learning by doing works well, it shows growth, confidence, and teamwork, but the whole discussion would feel more genuine if it spoke more from the participants’ perspective and less from a theoretical distance.

Thank you.

**Author Response:**
Thank you for this valuable feedback. We have revised the Discussion to foreground participants’ perspectives more clearly and to translate theoretical insights into concrete implications for training, supervision, and implementation practice. While theory remains an important interpretive lens, greater emphasis is now placed on how participants experienced and navigated the implementation process in practice.

**Reviewer #2:** Thank you for your great work.
I prefer to add some previous published researches whom used the QualCoder software in their works, and a little argument why you choose it specifically, is it for technical issues or else? For example other qualitative data analysis software offer some quireis which may be helpful for your study like Word Clouds; which is for to frequent words at the transcripts.
My suggestion is about quotes: if the data allow for choosing and using of single quote for each theme to be representative, it will be more informative, and due to reflexivity you can use the "big quote" to be rich in information related to the theme itself. Also, I suggest for more reflexive content to write about the prevalence, richness, and depth of themes; and use the result of these three parameters in your results, and discuss it at discussion section.

**Author Response:** Thank you for these helpful suggestions. We selected QualCoder for its open-source, transparent structure, which supports systematic documentation and continuity across first- and second-cycle coding conducted by different researchers. This rationale has now been clarified in the Methods section, and relevant literature has been added.

While some qualitative software offers frequency-based tools such as Word Clouds, our analytical focus was on interpretive depth rather than lexical frequency. In line with Reflexive Thematic Analysis (RTA), we prioritized meaning-making and contextual interpretation over visualization of word counts.

Regarding prevalence, richness, and depth, our analysis follows RTA principles, where frequency is not treated as an indicator of importance. Instead, analytical value is derived from conceptual coherence and interpretive richness. We have ensured that the Results and Discussion sections reflect the depth and complexity of themes accordingly, and the Methods section is revised to elaborate on reflexivity and development of themes.

We agree that illustrative quotes are important. To balance richness with clarity and journal word limits, we prioritized concise, context-rich quotes rather than extended verbatim excerpts, which can sometimes overshadow the analytical narrative. This approach aligns with the interpretive emphasis of RTA.

**Reviewer #3**: i have some minor suggestions:
1- Overly descriptive introduction: Could be shortened or restructured to emphasize the novelty and gap more directly.

**Author Response:** We appreciate this suggestion and have revised the introduction accordingly. Descriptive background content has been reduced, and the section has been restructured to foreground the research gap, novelty, and relevance of examining professionals’ experiences of guided digital care earlier and more clearly.

2- Method clarity: While reflexivity is well described, details on how saturation was assessed and how theme validity was verified could be expanded.

**Author Response:** We appreciate your suggestion to make it more focused and emphasize the novelty and research gap. We have revised the introduction accordingly by shortening the descriptive sections and restructuring the content to highlight the gap and contribution more clearly. We have clarified our analytic approach to align with Reflexive Thematic Analysis, where saturation is not treated as a guiding principle. We instead emphasize reflexivity, analytic depth, and coherence, and this has been clarified in the Methods section.

3- Discussion redundancy: Some subsections repeat points about training gaps and professional identity; could be tightened.

**Author Response:**
Thank you for this observation. We have revised the Discussion to reduce redundancy, streamline overlapping arguments, and improve conceptual clarity.

4- Formatting: Several typographical inconsistencies (extra spacing, line breaks, repeated author initials in the rigour section).

**Author Response:**
These issues have been corrected, and the manuscript has been carefully formatted.

5- Conclusion: Strong but could include explicit policy or training recommendations for wider implementation beyond Norway.

**Author Response:**
We have strengthened the Conclusion to include clearer implications for training and implementation strategies that are transferable beyond the Norwegian context.

Thank you again for helping us improve the quality of our work.
